# 异基因造血干细胞移植治愈不稳定血红蛋白病Hb Mizuho 1例

**DOI:** 10.3760/cma.j.issn.0253-2727.2023.06.018

**Published:** 2023-06

**Authors:** 婵 李, 高晖 杨, 练金 刘, 英华 陈, 雪梅 周, 永榕 赖, 容容 刘

**Affiliations:** 广西医科大学第一附属医院血液内科，南宁 530000 Department of Hematology, First Affiliated Hospital of Guangxi Medical University, Nanning 530000, China

患儿，男，8岁10个月，陕西西安人。患儿1岁时出现面色苍白、巩膜黄染，在当地医院检查发现黄疸、轻中度贫血，诊断溶血性贫血，原因未明确，未予特殊处理。为进一步诊治至我院就诊，血常规：WBC 8.65×10^9^/L，PLT 138.3×10^9^/L，RBC 3.12×10^12^/L，HGB 87.4 g/L，平均红细胞体积（MCV）88.07 fl，平均红细胞血红蛋白含量（MCH）28.05 pg，平均红细胞血红蛋白浓度（MCHC）318.50 g/L，网织红细胞绝对计数（Ret）0.37×10^12^/L。血生化：总胆红素（TBIL）102.1 µmol/L，间接胆红素（IBIL）95.2 µmol/L，血清铁蛋白（SF）203.79 µg/L，葡萄糖-6-磷酸脱氢酶（G-6-PD）活性正常。肽链分析：α链正常，β链减少，Gγ链++，Aγ链++。血红蛋白电泳：HbF 31.5％，HbA_2_ 2.1％。常见β地中海贫血基因分析正常。外周血涂片见成熟红细胞部分大小不等，可见椭圆形、泪滴形、棘形红细胞及红细胞碎片，较易见嗜多色、嗜点彩红细胞。未予特殊处理，患儿回到当地后予护肝等对症治疗。

此后患儿黄疸、贫血逐渐加重，HGB最低52 g/L，TBIL最高190.6 µmol/L，并出现肝脾肿大，于3岁时开始规律输血治疗，每20～30 d输血1次，每次输注红细胞1.5～2.0 U，维持HGB在80～100 g/L，未行祛铁治疗。5岁时再次来我院就诊，查体：贫血貌，骨骼发育无畸形，皮肤、眼结膜苍白，巩膜轻度黄染，脾肋缘下4 cm，肝脏肋缘下未触及。血常规：WBC 3.44×10^9^/L，PLT 111.9×10^9^/L，RBC 2.88×10^12^/L，HGB 85.7 g/L，MCV 88.99 fl，MCH 29.76 pg，MCHC 334.4 g/L，Ret 0.116×10^12^/L。血生化：TBIL 75.1 µmol/L，IBIL 67.4 µmol/L，SF 988.99 µg/L。血红蛋白电泳：HbF 8.0％，HbA_2_ 2.5％。异丙醇试验（−）。骨髓细胞形态学：红系增生显著，早幼及以下阶段细胞均见，部分胞质嗜多色，可见核固缩、花瓣核及不规则核，可见嗜碱性点彩晚幼红细胞，偶见双核，成熟红细胞大小不等，可见嗜多色性红细胞。珠蛋白基因Gap-PCR与高通量测序：未检测出α、β地中海贫血突变类型，检出Hb Mizuho［β68（E12）Leu→Pro（HBB:c.206T>C）］杂合突变。B超：脾大，脾门厚约4.2 cm，肋缘下平脐；肝回声未见异常。磁共振成像：心肌未见铁沉积（T2* 12.97 ms）；肝脏轻度铁沉积（1.8 mg/g干重）。诊断：不稳定血红蛋白病Hb Mizuho。随后行同胞全相合异基因造血干细胞移植，按白消安+环磷酰胺+氟达拉滨+抗胸腺细胞球蛋白方案预处理，分别回输供者骨髓、外周血干细胞采集物，共输入单个核细胞（MNC）18.94×10^8^/kg，CD34^+^细胞6.36×10^6^/kg，输注后予环孢素A+吗替麦考酚酯+甲氨蝶呤预防移植物抗宿主病（GVHD），低分子肝素+前列地尔预防肝静脉闭塞病（VOD），辅以预防感染、祛铁等治疗，术后有尿路感染，经治疗后好转，未发生GVHD、VOD，现已全部停药，HGB维持在正常水平，定期评估生长发育与同龄儿无显著差异。经家系调查，患儿无家族史，父母血常规无异常，珠蛋白基因测序均正常。

讨论：Hb Mizuho是一种罕见的不稳定血红蛋白，是β珠蛋白基因单个碱基突变（HBB:c.206T>C），使第68号密码子（E12）亮氨酸变为脯氨酸所致的。脯氨酸的插入破坏了正常E螺旋结构，从而扭曲β珠蛋白肽链的三级结构，最终导致Hb Mizuho不稳定。不稳定的Hb Mizuho易于变性和沉聚，使红细胞形变能力减弱而渗透性增强，引起慢性溶血性贫血。Hb Mizuho极度不稳定且氧亲和力异常升高，因此Hb Mizuho杂合的患者也可以表现出严重溶血性贫血。该病通常为获得性，表现为慢性溶血性贫血，常规检查与地中海贫血相似，由于病例缺乏，易被误诊。当考虑不稳定血红蛋白病时，可行热稳定性及异丙醇试验初筛，基因检测确诊。长期输血和祛铁是治疗本病的主要方法，异基因造血干细胞移植可治愈输血依赖的患者。

